# Early Home-Life Antecedents of Children’s Locus of Control

**DOI:** 10.3389/fpsyg.2018.02032

**Published:** 2018-11-01

**Authors:** Stephen Nowicki, Steven Gregory, Yasmin Iles-Caven, Genette Ellis, Jean Golding

**Affiliations:** ^1^Department of Psychology, Emory University, Atlanta, GA, United States; ^2^Centre for Academic Child Health, Bristol Medical School, University of Bristol, Bristol, United Kingdom

**Keywords:** ALSPAC, longitudinal cohort, child locus of control, parent locus of control, parent–child interaction

## Abstract

Children’s external locus of control has been linked to a wide variety of negative academic achievement, personality, and social adjustment outcomes. The purpose of this study was to discover which features of early home environment may facilitate the development of external as opposed to internal control expectancies in children. We use an exposome approach to analyze data from the Avon Longitudinal Study of Parents and Children (ALSPAC) cohort study, a longitudinal study starting in pregnancy in England in 1990–1992. Details of parents and their study children were collected prospectively, and children’s locus of control was assessed at age 8 using an abbreviated form of the most frequently used measure of children’s locus of control (Nowicki-Strickland Internal External locus of control scale). A series of stepwise logistic regression analyses were undertaken to determine the strongest independent associations. The final model (*n* = 4,075 children) comprised 13 variables – those with the strongest associations with the child becoming externally oriented were two that were positive indicators of the mother being distracted (TV on almost the whole time, and a consideration that pets should be treated as members of the family), three that were indicators of protective (negative) effects of interaction between mother and child (child was breast fed, mother read stories to the child, mother cuddled the baby when he/she woke at night), and two divergent indicators of maternal health behavior (more frequent cleaning of the child’s hands before a meal which was associated with a heightened risk of become external, and providing a healthy-type of diet, which was associated with a reduced risk of becoming external). The findings suggest that inadequate early maternal interaction with the child is associated with an increased risk of the child being externally oriented by the age of 8.

## Introduction

The purpose of the present study is to explore possible antecedents in the development of locus of control. Locus of control was introduced by Rotter (1966) and he defined it as follows. “Internal versus external control refers to the degree to which persons expect that a reinforcement or an outcome of their behavior is contingent on their own behavior or personal characteristics versus the degree to which persons expect that the reinforcement or outcome is a function of chance, luck, or fate, is under the control of powerful others, or is simply unpredictable. Such expectancies may generalize along a gradient based on the degree of semantic similarity of the situational cues.” (p. 1).

Rotter’s introduction of the locus of control construct stimulated the publication of thousands of studies (Nowicki and Duke, 1982, 2016; Nowicki, 2017) the results of which suggest being more, rather than less, externally controlled is associated with negative outcomes.

While a complete review of locus of control results is beyond the scope of the present paper, Crandall and Crandall (1983) summarized the considerable research results gathered in the two decades following the introduction of locus of control by Rotter:

“…perceptions of internal control, compared to perceptions of external control, are generally found to facilitate: (a) more active search of the environment for information relevant to salient goals, superior cognitive processing and recall of that information, and more incidental as well as intentional reaming; (b) more spontaneous engagement in achievement activities, selection of more challenging tasks, and better ability to delay gratification and to persist under difficulty; (c) higher levels of academic and vocational performance and more positive achievement-related attitudes; (d) more attempts to prevent and remediate health problems; (e) better interpersonal relationships, more assertiveness toward others, and more liking and respect from others, despite greater resistance to their influence; and (f) better emotional adjustment (higher self-esteem, better sense of humor, less anxiety, less depression, less severe psychiatric diagnoses, etc.) and greater reported life satisfaction and contentment.” (pp. 53–54).

More recent research results have provided support for Crandall and Crandall’s summary. More specifically, for example, higher external locus of control was associated with increased chances of being suicidal (Liu et al., 2005), depressed (Benassi et al., 1988; Luthar and Blatt, 1993; Garaigordobil et al., 2017), less persistent in completing tasks (McLeod, 1985), rejected by peers (Sandstrom and Coie, 1999), bullied by peers (Radliff et al., 2016), lonely (Doman and Roux, 2010), anxious (Rawson, 1992; Nanda et al., 2012; Ollendick and Grills, 2016; Chorpita et al., 2017), overly aggressive (Jung et al., 2018), academically less successful (Coleman et al., 1966; Kalechstein and Nowicki, 1997; Schelhas et al., 2012; Becares and Priest, 2015; Amata et al., 2017), lower in self-esteem (Wickline et al., 2011) and more anxious and depressed when suffering from Tourette’s syndrome (Cohen et al., 2008).

Because most research evidence suggests the disadvantage of being more rather than less external, the importance of knowing the antecedents that precede the development of external control expectancies is a priority.

### What Rotter and Others Suggested Were Antecedents of Locus of Control

Rotter (1966) assumed that learning “contingencies” between behavior and outcomes was at the heart of developing appropriate internality and theorized: (a) a generalized expectancy of internal control develops when reinforcement was perceived as contingent on the individual’s behavior; (b) once an expectancy is established, “reinforcement acts to strengthen an expectancy that a particular behavior or event will be followed by that reinforcement in the future and failure of the reinforcement to occur will reduce or extinguish the expectancy.” (p. 2). For Rotter, parents provide the primary source for contingency learning in their young children via “consistent discipline and treatment during their time together.” (p. 24).

Lefcourt (1976) also points out that parents are in a prime position to facilitate or hinder the contingency learning process. He believed warm supportive parents help to make children feel safe and secure enough to explore their environments and learn how their behavior connects to outcomes across a variety of situations; internal control expectancies can develop and generalize from such experiences. In contrast, parents who neglect or reject their children may be more likely to produce anxiety that interferes with learning connections between behavior and outcomes and constricts the number and quality of children’s interactions with the physical and social environments.

For the most part, research is supportive of Rotter and Lefcourt’s views. Carton and Nowicki (1994) reviewed the antecedent literature and concluded that: “Children with generalized internal, as opposed to external, control expectancies report less stress earlier in their lives and have parents who report treating them more consistently, granting them greater autonomy to pursue their activities earlier, and providing them with a warm, supportive relationship.” (p. 139).

However, Carton and Nowicki (1994) go on to criticize the field’s use of cross-sectional, self-report methodologies with small samples of non-representative participants and suggested large scale prospective cohort approaches with representative populations could obtain more valid data to support or refute what has been found previously regarding antecedents of locus of control. The present paper is an attempt to carry Carton and Nowicki’s recommendation forward.

### The Present Study

Early experiences in the home may set the tone for children’s ability to pick up the relevance between their behavior and outcomes. Past research has typically used retrospective or correlative methodologies to gather general information about parent disciplinary practices or children’s view of parents’ actions. Few have looked at the specific everyday activities within the home, especially before the age of five, to get a sense of the “nuts and bolts” of the typical home environment’s ability to favor or hinder behavior, outcome contingency learning. While maternal factors will be a primary focus, we use an exposome approach to uncover relevant factors associated with children’s locus of control prior to their fifth birthday.

It should be noted that the data presented form a unique resource in that they are prospectively collected from a large population cohort which has measured the locus of control of both parents and children over time. An additional advantage concerns the large number of different variables collected which measure the domestic environment and parenting behaviors.

## Materials and Methods

### Participants

The Avon Longitudinal Study of Parents and Children (ALSPAC) is a pre-birth cohort designed to determine the environmental and genetic factors associated with the development and health of the children born to the women recruited in pregnancy (Golding and Alspac Study Team, 2004; Boyd et al., 2013; Fraser et al., 2013). As part of the study design there was a concerted effort before the child’s birth to obtain from the parents details of their personalities, moods and attitudes, including a measure of their locus of control (LOC). LOC scores were then measured in the children at ages 8 and 16.

ALSPAC recruited 14,541 pregnant women residents in Avon, United Kingdom with expected dates of delivery between 1st April 1991 and 31st December 1992. This was an estimated 80% of the eligible population. Of these initial pregnancies, there was a total of 14,676 fetuses, resulting in 14,062 live births, 13,988 of whom were alive at 1 year of age. Data were collected at various time-points via self-completion questionnaires, biological samples, hands-on measurements, and linkage to other data sets. Please note that the study website contains details of all the data that is available through a fully searchable data dictionary and variable search tool: http://www.bristol.ac.uk/alspac/researchers/our-data/.

Ethical approval for the study was obtained from the ALSPAC Ethics and Law Committee (ALEC) [ALEC; IRB00003312] (registered on the Office of Human Research Protections database as UBristol IRB #1) and the Local Research Ethics Committees. ALEC agreed that consent was implied if questionnaires were returned. Informed written consent was obtained for all biological samples prior to analysis, and for certain invasive procedures during the hands-on assessments. The hands-on assessments carried out on a random 10% of the children aged <5 years and the full cohort at ages 7 onward were optional to attend and each individual measure in these clinics was also optional and only carried out with signed consent from a parent and assent from the child (Birmingham, 2018).

Following advice from ALEC, the fathers were not enrolled directly, but involved by sending questionnaires to the mothers to pass on to them if they wished, with a separate reply-paid envelope. The study deliberately had no information on whether the mother had invited her partner to take part except when the completed questionnaire was returned. In consequence of this protocol, there was no way in which the study could send reminders directly to the partners. However, 76% of the partners returned their questionnaires during the pregnancy.

The study population has been shown, by comparison with the 1991 Census, to be approximately representative of the area, and of the United Kingdom population in general. It should be noted, however, that at this time the proportion of non-white children in England was low (∼6%), and was only ∼5% of ALSPAC children. Just 5% of mothers were teenagers at the time of birth, and a further 19% were under 25; 10% were 35 or over. The proportion of mothers with University degrees was 14%, and a further 14% had very low or no scholastic qualifications. The study area included the city of Bristol as well as smaller urban and semi-urban areas, as well as rural communities.

### Outcome Measure

The children’s locus of control measure used in the present study was an adaptation of the Children’s Nowicki Strickland Internal External scale (CNSIE, Nowicki and Strickland, 1973) The CNSIE has been used in hundreds of studies that have provided data supportive of their construct validity. The full CNSIE, comprising 40 questions, was administered to a sample of 120 eight-year-old children and the 12 items with the best item-total correlation were chosen for inclusion in the final form. This form was administered to ALSPAC children when they were tested at 8 years of age. Examiners read LOC questions out loud to the child (to control for reading ability) as part of the tests that were undertaken in a clinic specially designed to test the children physically and psychologically. The child was asked to respond with a yes/no answer. It was made clear by the tester that there were no right or wrong answers and that we were just interested in knowing how different people think and feel about different things; in addition, the children were reminded that their answers were confidential.

### The Exposome Strategy

We first use a hypothesis-free approach to identify factors that are associated with childhood externality by assessing the association of this binary variable with 1355 variables associated with exposure of the child in the preschool phase (<5 years). These include facets of labor and delivery (35 variables), diet and nutrition (532), sleeping conditions (41), aspects of child care and parenting (155), equipment and toys available (49), social and housing circumstances (362), pets, pests and hygiene (112), life events to the child (44) and other miscellaneous features (25). The variables are frequently not independent of one another: for example, the same exposure may be considered at several different ages. The following strategy was therefore used: (a) unadjusted associations with *P* < 0.0001 were selected (*n* = 247); (b) those variables measuring similar features were examined and a selection was made based on the validity of the variable, the amount of missing data and the effect sizes. The full set of 1,355 associations with odds ratios and 95% confidence intervals are available from the corresponding author.

The remaining variables are described in the Appendix. They were arbitrarily formed into three logical groups as described in the Results section. First backward stepwise logistic regression was used with each group to identify which variables were independently associated with the child having an external LOC. Finally, the independent factors within each group were combined and a further backward logistic regression was undertaken to determine a final model. Comparisons of the goodness of fit (GOF) employed the pseudo *R*^2^ statistic, the higher the value the better the fit.

## Results

**FIGURE 1:**
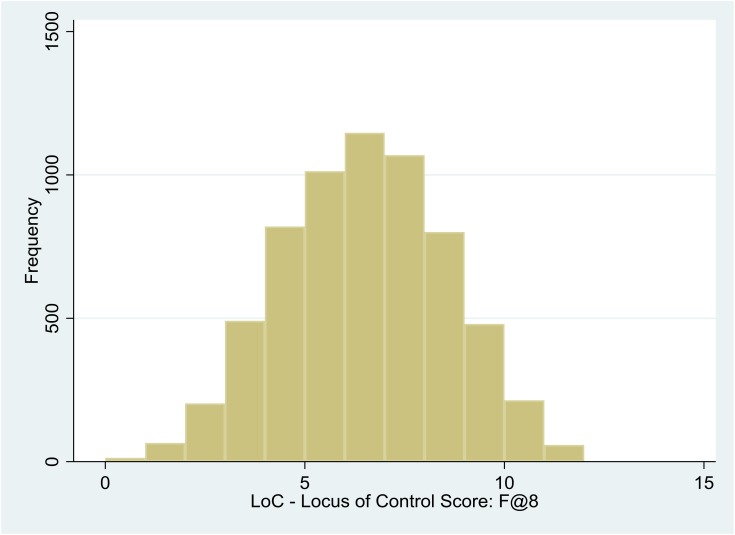
Histogram showing the distribution of children’s locus of control scores at age 8.

The 12 questions comprising the child’s LOC score were completed by 6,381 of the 8-year-old children. The distribution of the LOC scores was approximately normal (Figure 1), with median of 6 and 41.2% defined as External (i.e., score > median).

### Early Home Environmental Experiences

The 11 dominating environmental variables that were associated with the child becoming external are shown in Table 1. The children living in public housing and those in homes with over-crowding were more likely to become external, as were those where the children were frequently in rooms where people were smoking. Unexpectedly there were also positive associations with the presence of a pet in the home, especially of a dog; the mothers who declared that pets should be treated as members of the family were more likely to have a child with an external orientation. Conversely, those whose chief caretaker had changed between ages of 18 and 30 months were more likely to be internal, but those who had their hands cleaned before a meal and those who did not swallow toothpaste were more likely to be external. Finally, there were two variables denoting television in the home: where there was a TV on most of the time, the child was more likely to become external, and there was a strong trend with the amount of time the child watched television at age 3, the more time spent watching the more likely to be external.

**Table 1:** Variation in proportion of children who became external according to early home environment experiences.

	Proportion (*n*) who became external	*p*
*Lives in public housing at 8 months*		
Yes	54.2% (272)	
No	38.9% (2228)	<0.0001
*Crowding (persons/room) at 21 months*		
<0.5	33.8% (425)	
0.5–0.99	40.7% (1344)	
1.00+	44.8% (546)	<0.0001
*Passive smoke exposure at 15 months*		
Yes	46.7% (1045)	
No	36.6% (1455)	<0.0001
*Pets in home at 8 months*		
Yes	42.8% (1428)	
No	37.6% (1086)	<0.0001
*Dog in home at 8 months*		
No	39.0% (1949)	
Yes	45.9% (565)	<0.0001
*Mother believes pets should be treated as family*		
Yes	49.3% (578)	
No	37.7% (1717)	<0.0001
*Child cleans hands before meals at 15 months*		
Always	46.5% (329)	
Usually	41.7% (916)	
Sometimes	38.2% (727)	
Occasionally	38.2% (393)	
Never	35.4% (127)	<0.0001
*Child swallows toothpaste at 2 years*		
Yes	37.0% (1244)	
No	43.6% (1124)	<0.0001
*TV on most of the day at 30 months*		
Yes	46.7% (1240)	
No	35.1% (954)	<0.0001
*Time child spent watching TV per week at 3 years*		
0–3 h	32.8% (67)	
3.5–6 h	36.0% (705)	
6.5–9 h	40.6% (986)	
>9 h	46.3% (630)	<0.0001
*Changed carer between ages 18 and 30 months*		
Yes	28.8% (127)	
No	41.2% (2297)	<0.0001

The 11 variables were offered to a stepwise logistic regression analysis – three dropped out: the crowding variable at 21 months, whether there were pets in the home at 8 months, and the amount of time the child watched TV at 3 years (Table 2).

**Table 2:** Aspects of early child’s social conditions related to the child having an external LOC at age 8: all children.

Social conditions	Unadjusted			Adjusted		
	*n*	OR [95% CI]	*p*	*n*	OR [95% CI]	*p*
Council accommodation 8 months	6219	1.86 [1.54, 2.29]	<0.001	4606	1.38 [1.09, 1.75]	0.008
Persons/room 21 months	5770	1.15 [1.09, 1.21]	<0.001		–	
Exposed to passive smoke 15 months	6211	1.51 [1.36, 1.68]	<0.001	4606	1.29 [1.13, 1.46]	<0.001
Household owned pets 8 months	6222	1.24 [1.12, 1.37]	<0.001		–	
Owned a dog	6222	1.33 [1.17, 1.51]	<0.001	4606	1.24 [1.06, 1.44]	0.006
Mother belief in pets as family	5722	1.37 [1.27, 1.47]	<0.001	4606	1.20 [1.10, 1.32]	<0.001
Child’s hands cleaned before meals	6192	1.17 [1.09, 1.25]	<0.001	4606	1.11 [1.03, 1.21]	0.010
Child swallows toothpaste	5938	0.76 [0.68, 0.84]	<0.001	4606	0.85 [0.75, 0.97]	0.012
TV on most of the day 30 months	5610	1.65 [1.48, 1.84]	<0.001	4606	1.54 [1.36, 1.74]	<0.001
Time child spent watching TV 3 years	5953	1.05 [1.03, 1.06]	<0.001		–	
Changed carer from 18 to 30 months	6014	0.58 [0.47, 0.72]	<0.001	4606	0.71 [0.57, 0.91]	0.006

*GOF = 2.89*.

### Early Parenting

There were nine parenting variables with strong associations with subsequent risk of the child having an external orientation (Table 3): only two had a positive association however (a grand-parent looking after the child, and the mother slapping him/her were both associated with the child being external). The apparently protective factors were cuddling the baby when awake in the night; using a baby sling at 8 months; having paid child care; taking the child to a library or place of interest at 30 months; singing and/or reading to the child at 2 years. Stepwise logistic regression analyses using these nine variables resulted in three dropping out: paid child care; the mother singing and the child going to a library (Table 4).

**Table 3:** Variation in proportion of children who became external according to early parenting experiences.

	Proportion (*n*) who became external	*p*
*Baby was cuddled when woke at night*		
Always	35.5% (517)	
Usually	38.8% (380)	
Sometimes	43.1% (868)	
Never	41.7% (613)	<0.0001
*Grandparent looked after child at 8 months*		
Yes	43.2% (1227)	
No	37.7% (1250)	<0.0001
*Paid child care at 8 months*		
Yes	33.3% (256)	
No	41.2% (2221)	<0.0001
*Child is taken to a library at 30 months*		
Yes	35.4% (827)	
No	43.3% (1582)	<0.0001
*Child taken to places of interest at 30 months*		
Yes	36.2% (1202)	
No	45.2% (1214)	<0.0001
*Mother sings to child every day*		
Yes	38.2% (1406)	
No	44.9% (1030)	<0.0001
*Child is read to by mother every day*		
Yes	35.4% (1377)	
No	48.5% (1051)	<0.0001
*Mother slaps child*		
1+ times/week	44.3% (785)	
Rarely	40.3% (1183)	
Never	34.6% (463)	<0.0001
*Mother used baby sling at 8 months*		
Yes	36.2% (1168)	
No	44.6% (1356)	<0.0001

**Table 4:** Aspects of early child’s parenting related to the child having an external LOC at age 8: all children.

Aspects of parenting	Unadjusted			Adjusted		
	*n*	OR [95% CI]	*p*	*n*	OR [95% CI]	*p*
Cuddles baby when wakes at night	5918	0.86 [0.81, 0.92]	<0.001	4690	0.89 [0.83, 0.95]	0.001
Grandparent looks after child 8 months	6149	1.26 [1.14, 1.39]	<0.001	4690	1.15 [1.02, 1.30]	0.022
Paid childcare at 8 months	6150	0.71 [0.61, 0.84]	<0.001		–	
Child goes to library 30 months	5990	0.85 [0.81, 0.89]	<0.001		–	
Visits to places of interest 30 months	6010	0.79 [0.74, 0.84]	<0.001	4690	0.92 [0.85, 0.99]	0.029
Mother sings to child daily 2 years	6068	0.83 [0.74, 0.92]	<0.001		–	
Stories read to child at 2 years	6056	0.74 [0.69, 0.79]	<0.001	4690	0.80 [0.74, 0.86]	<0.001
Child slapped by mother 2 years	6044	1.18 [1.12, 1.24]	<0.001	4690	1.08 [1.01, 1.14]	0.023
Mother uses baby sling at 8 months	6245	0.70 [0.64, 0.78]	<0.001	4690	0.86 [0.76, 0.98]	0.020

*GOF = 2.59*.

### Dietary Practices

In all, 11 variables related to diet or related behaviors (Tables 5A,B). Those that were associated with the child being more likely to be internal were: the length of time before the newborn baby was put to the breast; whether breast fed in the first month of life; whether fed on demand; whether being given a traditional type of diet; and/or one that used foods assumed to be healthy. Conversely, the following were predictive of the child being external: the mother stating that she had loved the baby immediately; the child being given a dummy (comforter or pacifier) in the first month of life; having formula by 6 months of age; having tea and/or cola to drink regularly in the period 6–15 months; and having a diet largely dominated by processed foods.

**Table 5A:** Variation in features of feeding of infant and child with their subsequent LOC at age 8.

	Proportion (*n*) who became external	*p*
*Time after delivery until first put to breast*		
<1 h	36.9% (1333)	
1+ h	41.3% (685)	
Not put to breast	51.0% (541)	<0.0001
*Mother loved baby immediately*		
Yes	41.6% (2073)	
No	35.9% (480)	<0.001
*Any breast feeding in the first month*		
Yes	38.3% (2052)	
No	51.3% (554)	<0.0001
*Fed on demand in the first month*		
Yes	38.0% (1756)	
No	47.0% (840)	<0.0001
*Baby used a dummy in first 4 weeks*		
Yes	43.6% (1549)	
No	36.6% (1057)	<0.0001
*Given formula in first 6 months*		
Yes	41.6% (2107)	
No	35.0% (438)	<0.0001
*Drank tea 6–15 months*		
Yes	46.0% (1033)	
No	37.0% (1460)	<0.0001
*Had cola 6–15 months*		
Yes	47.5% (534)	
No	38.7% (1957)	<0.0001

**Table 5B:** Mean difference in scores [95% CI] of dietary patterns at 2 years (Northstone and Emmett, 2013) in those who became external compared with those who were internal.

	95% CI	*p*
Traditional	-0.15 [-0.20, -0.10]	<0.0001
Processed	+0.18 [0.13, 0.23]	<0.0001
Healthy type	-0.22 [-0.28, -0.17]	<0.0001

When these 11 variables were offered to stepwise logistic regression, those that dropped out were being breast fed in the first month; being fed on demand, having formula by 6 months of age and being given cola to drink in the period from 6 to 15 months of age (Table 6).

**Table 6:** Aspects of early child diet related to the child having an external LOC at age 8: all children.

Child’s diet	Unadjusted			Adjusted		
	*n*	OR [95% CI]	*p*	*n*	OR [95% CI]	*p*
Time before put to breast	6339	1.31 [1.23, 1.40]	<0.001	5326	1.18 [1.09,1.27]	<0.001
Mother loved immediately	6323	1.27 [1.12, 1.43]	<0.001	5326	1.22 [1.06, 1.40]	0.006
Breast fed in first month	6442	0.59 [0.52, 0.67]	<0.001		-	
Fed on demand	6413	0.69 [0.62, 0.77]	<0.001		-	
Used a dummy in first month	6442	1.33 [1.21, 1.48]	<0.001	5326	1.20 [1.07, 1.35]	0.002
Given formula by sixth month	6313	0.76 [0.66, 0.86]	<0.001		-	
Drank tea between 6 and 15 months	6190	1.45 [1.30, 1.61]	<0.001	5326	1.21 [1.07, 1.37]	0.003
Drank cola between 6 and 15 months	6187	1.43 [1.26, 1.63]	<0.001		-	
Had traditional diet aged 2 years	6753	0.85 [0.81, 0.90]	<0.001	5326	0.87 [0.83, 0.93]	<0.001
Diet of mostly processed food 2 years	6753	1.22 [1.15, 1.29]	<0.001	5326	1.14 [1.07, 1.21]	<0.001
Healthy-type diet at 2 years	6753	0.80 [0.75, 0.84]	<0.001	5326	0.84 [0.79, 0.89]	<0.001

*GOF = 2.49*.

### The Final Model

Offering the variables that survived each of the inter-group analyses (Tables 2, 4, 6) to a stepwise regression, the following eight variables dropped out: living in public housing, child swallowing toothpaste, and changed carer between 18 and 30 months, mother used a baby sling at 8 months, grandparents look after the child at 8 months, child is taken to places of interest at 30 months, mother said that she loved the child immediately after the birth, and that she used a dummy in the first month of life. Thirteen variables comprised the final model (Table 7). Of these, those that were particularly strong (arbitrarily defined as *P* < 0.01) were the following predictors of externality in the child: maternal belief that pets should be treated as members of the family; frequency with which the child’s hands are cleaned before a meal; the television being on for all or most of the day; and the fact that the newborn baby was never put to the breast. Strong protective factors were the mother cuddling the baby when he/she woke at night; reading to the child at age 2; and having a healthy-type of diet at 2 years.

**Table 7:** The final model predicting the externality of the child at age 8.

Variable	AOR [95% CI]	*p*
*Social conditions*		
Exposed to passive smoke 15 months	1.17 [1.01, 1.34]	0.034
Owned a dog	1.18 [1.00, 1.39]	0.046
Mother belief in pets as family	1.19 [1.08, 1.30]	<0.001
Child’s hands cleaned before meals	1.16 [1.06, 1.26]	0.001
TV on most of the day 30 months	1.37 [1.20, 1.57]	<0.001
*Parenting*		
Cuddles baby when wakes at night	0.90 [0.83, 0.97]	0.006
Stories read to child at 2 years	0.86 [0.79, 0.93]	<0.001
Child slapped by mother 2 years	1.08 [1.01, 1.16]	0.031
*Feeding and diet*		
Baby never put to breast	1.13 [1.04, 1.24]	0.007
Drank tea between 6 and 15 months	1.17 [1.01, 1.35]	0.038
Had traditional diet aged 2 years	0.91 [0.85, 0.98]	0.010
Diet of mostly processed food 2 years	1.09 [1.01, 1.18]	0.023
Healthy-type diet at 2 years	0.90 [0.84, 0.97]	0.004

*GOF = 4.20; *N* = 4075. AOR, adjusted odds ratio; CI, confidence interval*.

## Discussion

While in some ways parent locus of control might have been expected to be a relevant antecedent of children’s orientation, past research suggests otherwise. A previous study using ALSPAC data found correlations between parent and child locus of control ranged from 0.14 to 0.19. (Nowicki et al., 2018b) The low parent, child correlations suggest other factors are at play in developing children’s locus of control orientations especially during their early years spent largely in the home. In order to investigate what factors may be having such an influence this study has used a hypothesis free approach to uncover features of the child’s first 5 years of life associated with him/her being more externally oriented at 8 years of age. As suggested by the literature, indicators of a warm nurturing background (breast feeding; cuddling when waking at night; being read stories) were related to being less external, whereas indicators of maternal interests such as being less focused on the child (having the TV on almost all day; believing pets should be treated as equal parts of the family) were positively associated with the child being more external.

Rotter (1966) and Lefcourt (1976) theorized that greater maternal distance and lack of attention would hinder children’s ability to learn which aspects of their behavior were contingent with what happens to them and, in essence, that is what we found in the present study. External mothers showed indicators that they were less likely to develop close and nurturing relationships with their children (for example, putting family pets on an equal footing with their children in terms of attention). Our methodology did not allow us to observe what actually occurred during the interactions between mothers and their young children, however the findings of children having reduced time with and attention from their mothers is consistent with the idea that less contingent learning may be taking place which in turn may produce greater externality. This possibility is supported by the work of Carton et al. (1996). They observed the interactions between mothers and their children on a series of puzzle tasks and found that mothers of externals were more likely to be more intrusive, interfering and off-task than mothers of internals. Additional research is needed to more fully understand what is happening during mother, child interactions not only in the lab, but in the familiarity of their home settings.

### Difficulties in Measuring Antecedents

Identifying other antecedents of locus of control besides parent orientations is more difficult than finding antecedents of traditional personality traits. For one thing, although generalized expectancies tend to become more stable with age, they are fluid and open to change in reaction to environmental and behavioral events throughout life. For example, we found that changes in parent orientations were associated with changes in personal relationships and economic events that may also impact on their children (Nowicki et al., 2018a).

For another thing, antecedents of locus of control can change depending on the age and social situation of the child (Nowicki, 2016). If children are in a “fair” environment, one that allows them to learn contingencies between behavior and outcomes, their increasing ability to impact on their environment will lead them to become more internal with age (see Nowicki and Strickland, 1973).

For a third thing, there is no absolute cut off score to determine who is internal or external. Most often, researchers decide internality and externality statistically *post hoc* by dividing the test distribution at an arbitrary point such as the mean or median or into extremes like top third and bottom third. In rare instances, enough individuals are tested at an age to enable the use of scores that reflect something approaching norms (Nowicki, 2017).

The ALSPAC and the 1970 British national birth cohort data sets contain a substantial number of children for whom LOC scores are available and it has allowed us to determine the contribution of child’s LOC to a variety of specific outcomes such as obesity in adolescence (Golding et al., 2018) and adulthood (Gale et al., 2008), poor self-rated health at age 30 (Gale et al., 2008), and with poor academic outcomes (Flouri, 2006), thus providing the measure with construct validity.

### Strengths and Weaknesses of the Study

Thus, the strengths lie in the large sample size, the population selection, and the fact that detailed features of childhood were collected from pregnancy onward (and thus independent of the child outcome considered). This is the first large prospective longitudinal study to use an exposome type of analysis to determine features of the child’s early upbringing in the home that are independently associated with the development of externality in 8-year-old children. As expected it has identified two aspects of the child’s life that have not been considered before but fit within the theoretical framework – both are indicators of the child being less than central to the mothers’ attention (having a television on almost the whole of the day and the mother having a strong relationship with household pets).

The weaknesses concern the fact that those parents who were externally oriented were less likely to bring their child for examination (and hence less likely for the child to complete the locus of control scale). There is no means of knowing whether their exclusion is more likely to have diminished the true associations rather than exaggerated them. One other possible weakness concerns the fact that we have only considered environmental factors that were present during early childhood, and it may be that influences in early school life may also have influenced the development of the child’s LOC. Finally, although this is a substantial population study, with all social strata represented, it is unclear as to whether our results can be translated to a different population or even to a different time period – thus our results need replicating.

## Conclusion

Evidence is substantial for the association of locus of control with an impressive array of important child outcomes varying from academic achievement to psychological adjustment. Within different age groups, greater externality appears to be associated with more negative outcomes for children. Finding out what may lead some children to be more external than their peers is useful in understanding the dynamics of the acquisition of both external and internal expectancies and in potentially developing interventions to facilitate the growth of appropriate internal expectancies during early childhood. However, there is a lack of concrete information about which aspects of mother–child interactions during preschool years facilitate or hinder the contingency learning so necessary for the development of appropriate internal expectancies. The present study identified some significant mother, child behaviors that were associated with externality in the child. Hopefully future researchers will use other populations to see if these aspects of children’s early years in the home are associated with the development of internal and external expectancies.

## Author Contributions

JG planned and carried out the analyses with SG and GE. SN and JG wrote the first draft of the manuscript. All authors were involved in editing, checking, and rewriting the paper.

## Conflict of Interest Statement

The authors declare that the research was conducted in the absence of any commercial or financial relationships that could be construed as a potential conflict of interest.

## Appendix

Here, we describe the variables used in the analyses in this paper, with their background where appropriate. All were selected based on their unadjusted associations with the child’s externality at age 8 (see the section “Materials and Methods” for selection criteria).

***Early Home Environmental Experiences***

(i) Council accommodation (i.e., public housing) - This variable was selected from the question asked on the tenure of the housing in which the child resided. This was asked at 8, 21, 33, and 61 months. The variable at 8 months was selected.

(ii) Crowding: This was calculated as the number of persons in the household divided by the number of rooms used for living or sleeping - this excluded kitchens too small to eat in, bathrooms, etc. The variable was available for 21 and 33 months, and the 21-month variable was selected.

Exposure to passive smoking: This variable was in answer to the question: ‘Please indicate how often during the day he/she is in a room or enclosed place where people are smoking. The mother was asked separately for weekdays and weekend days, and was given the following options: ‘all the time; more than 5 h; 3, 4 or 5 h; 1 or 2 h; less than 1 h; not at all. For this study no exposure was coded if both the weekdays and weekend days had indicated ‘not at all.’

Pets in the household: The mother was asked approximately annually whether there were any pets in the household. Here, we took the answers given when the child was 8 months old.

Dog(s) in the household: This was a supplementary question following the question on pets.

Attitude of mother toward pets: When the child was 33 months old the mother was asked her reaction to the statement: ‘Pets should have the same rights and privileges as family members.’ And was given the possible responses: ‘Strongly agree; Agree; Disagree; Strongly disagree.’ Here, the first two and last two responses were combined.

Hands cleaned: When the child was 15 months old, the mother was asked - ‘All children get dirty. How often in a normal day . are her hands cleaned before a meal?’ Possible responses were: always; usually; sometimes; occasionally; never. For the analyses the third and fourth groups were combined.

Swallows toothpaste: At 2 years a question concerned whether when cleaning his/her teeth the child swallowed the toothpaste or spat it out. The options were: swallows it; spits it out; varies. Here, the answers were dichotomized into those who consistently spat it out and the rest.

Television in the home: There were two questions on the TV that were used. (i) At 30 months the mother was asked: ‘When do you have the television on?’ and given the options ‘all day; most of the day; mornings only; afternoons only; evenings only; not at all; do not have a TV. For these analyses ‘all day’ and ‘most of the day’ were combined. (ii) At 3 years she was asked how much time the child spent watching TV per week.

Changed carer: This question was part of a series of potentially life changing events occurring between 18 and 30 months.

***Early Parenting Strategies***

Cuddling of baby: At 6 months the mother was asked what she did when the baby woke at night. Among the options she was asked how frequently she rocked or cuddled him/her, and given the options: Always; Usually; Sometimes; Never.

Grandparent looked after the child: At 8 months the mother was asked: ‘Apart from yourself, who regularly looks after your baby when you are out?’ One of the options was the baby’s grandparent.

Paid child care: Another option at 8 months was: paid person outside baby’s home (e.g., child minder).

Taken to library: At 30 months the mother was asked: ‘How often do you take him/her to . a library’ with possible options: ’nearly every day; about once a week; once a month; a few times a year; never.’ Here, we have combined once a month or more frequently as ‘yes’ and less than once a month or never as ‘no.’

Taken to places of interest: This was part of the same set of questions at 30 months, and the same categorization was used.

Activities with the child: When the child was 2 years, the mother was asked When you are at home with your child, how often do you do the following: and was given the responses: Every day; several times a week; about once a week; rarely; never. Among the activities, was: (i) Sings to child; (ii) Read him/her stories; (iii) Slaps him/her.

Use of baby sling: At 8 months, the mother was asked whether she had a Sling or back pack for carrying the child and was given the possible responses: Yes but not used; yes and used; no, do not have. In this study we distinguish between those who had and used their sling from the remainder.

***Dietary Practices:*** In this section, we include dietary factors as well as two factors that were not strictly dietary (use of dummy/comforter and time taken to love the baby). The actual items used are as follows.

Time after delivery until first put to the breast: This is a measure of breast feeding as well as of the time to the initiation of breast feeding.

Time to love baby: At 4 weeks the questionnaire stated - ‘Often mothers are surprised how long it takes to love their babies. How long has it taken you?’ The possible responses were: ‘I loved him immediately; It took a little while; It took over a week; I still do not love him fully; can’t remember.’ Here, we compared those who said they loved the baby immediately with the remainder.

Breast fed: This variable considered any breast feeding in the first month of life.

Fed on demand: The mother was asked at 4 weeks - ’Is your baby fed (either by breast or bottle) on a regular schedule (e.g., every 4 h)? And was given the options: yes always; yes try to; no, fed on demand.

Baby used a dummy: At 4 weeks the mother was asked for daytime and for the night - ’Does your baby have a dummy or comforter? Possible responses were: Usually; Often; Sometimes; Never. The variable distinguished the babies that were given a dummy (comforter/pacifier) whether by day or night from those who never used a dummy.

Formula at 6 months: The mother was asked whether the baby had had a bottle of formula by age 6 months.

Tea: At 15 months the mother was asked whether the toddler had had tea to drink.

Cola: At 15 months the mother was asked whether the toddler had had cola to drink.

Dietary patterns: At age 2 years the frequencies of foods and drinks which the child was consuming were used to identify three types of dietary pattern (Nanda et al., 2012):

(i) Traditional diet also called ‘family foods.’ This was a score that particularly identified traditional British family foods such as meat, fish, puddings, potatoes, and vegetables.

(ii) Processed foods: Many of the food loadings on this pattern were high in sugar such as sweets, chocolate, fizzy drinks, and flavored milks. Other foods associated with this pattern such as crisps, potatoes, baked beans, peas, and soup were foods required little in the way of cooking.

(iii) Healthy-type: This pattern was loaded with foods often recommended as the basis for a healthy diet, such as fruit, vegetables, eggs, nuts, and juices.
